# Supplementary material for: Sustained-input switches for transcription factors and microRNAs are central building blocks of eukaryotic gene circuits
Source: Genome Biol. 2013 Aug 23;14(8):R85. doi: 10.1186/gb-2013-14-8-r85 (PMC4054853; doi:10.1186/gb-2013-14-8-r85)
Supplement: Additional file 5 — HTML Browsable Motif Output. Zipped folder containing all WaRSwap and FANMOD motif output, viewable in a web browser. [file gb-2013-14-8-r85-S5.ZIP › HTML_browsable_motif_output/FANMOD_ath_tair9/sigs_fanmodm-2000.pvals.heatmaps.html/motif_id_46_000101110_tftype_ath_upstream_-2000_0.html]

```
BG_MODEL = FANMOD
MOTIF_ID = 46_000101110
TF_TYPE = ath
UPSTREAM = -2000_0


PVals
FN_0.2	FN_0.4	FN_0.6	FN_0.8
dg_60.genes	0.651	0.001	0.083	0.458
dg_70.genes	0.653	0.001	0.102	0.444
dg_80.genes	0.654	0	0.079	0.457

ZScores
FN_0.2	FN_0.4	FN_0.6	FN_0.8
dg_60.genes	-0.378	3.22	1.342	-0.759
dg_70.genes	-0.4	3.146	1.239	-0.745
dg_80.genes	-0.388	3.407	1.339	-0.767

StDevs
FN_0.2	FN_0.4	FN_0.6	FN_0.8
dg_60.genes	33.384	26.858	11.311	1.479
dg_70.genes	33.303	27.68	11.844	1.464
dg_80.genes	32.463	25.72	11.091	1.451
```
